# Quality Control of PET Bottles Caps with Dedicated Image Calibration and Deep Neural Networks

**DOI:** 10.3390/s21020501

**Published:** 2021-01-12

**Authors:** Marcin Malesa, Piotr Rajkiewicz

**Affiliations:** KSM Vision sp. z o.o., ul. Sokołowska 9/117, 01-142 Warsaw, Poland; p.rajkiewicz@ksmvision.pl

**Keywords:** machine vision, quality control, neural networks, image processing

## Abstract

Product quality control is currently the leading trend in industrial production. It is heading towards the exact analysis of each product before reaching the end customer. Every stage of production control is of particular importance in the food and pharmaceutical industries, where, apart from visual issues, additional safety regulations are demanded. Many production processes can be controlled completely contactless through the use of machine vision cameras and advanced image processing techniques. The most dynamically growing sector of image analysis methods are solutions based on deep neural networks. Their major advantages are fast performance, robustness, and the fact that they can be exploited even in complicated classification problems. However, the use of machine learning methods on high-performance production lines may be limited by inference time or, in the case of multiformated production lines, training time. The article presents a novel data preprocessing (or calibration) method. It uses prior knowledge about the optical system, which enables the use of the lightweight Convolutional Neural Network (CNN) model for product quality control of polyethylene terephthalate (PET) bottle caps. The combination of preprocessing with the lightweight CNN model resulted in at least a five-fold reduction in prediction and training time compared to the lighter standard models tested on ImageNet, without loss of accuracy.

## 1. Introduction

Food and drink manufacturers are obliged to control the quality of their products (regulatory issues). The release of defective products (and/or packages) may result in complaints or even the return of a whole series of products (especially in the food and pharmaceutical industries). To reduce the risk of financial and reputational loss, manufacturers install systems to control all their products [1].

A widely used approach to quality control is applying systems based on machine vision and image processing [2,3,4]. The basic advantages of those solutions, such as efficiency, lack of contact, or a relatively low degree of complexity, make them a frequent choice for quality control in the food industry [5,6]. Image-based systems can only be used when it is possible to distinguish defective and acceptable products based on images (in some electromagnetic wave range: ultraviolet, visible light, infrared, and X-ray). At the same time, the quality control system must cover the entire area where the defect may occur, which in some cases may be difficult due to the size or geometry of the tested objects. A frequently used solution is to multiply cameras observing the object from different sides to register the entire surface of the object [7,8]. However, it may make the measurement system more complicated and the development of algorithms for automatic image analysis may be much more difficult as the views from different cameras may differ from each other. The applicability of such systems in the same form and with the same image processing algorithms on a different production line would be limited. Further, the analysis of several images of the same object increases the requirements for computing power, which, as a consequence, may limit the production efficiency (which is unacceptable from the manufacturer’s point of view) or significantly affect the cost of the system itself.

The architecture of the visual quality control system can be simplified by using knowledge about the tested product and an intelligent combination of hardware and software solutions. In hardware solutions, the design of the optical system is crucial. It has to simplify the construction and at the same time facilitate the automation of image processing and classification algorithms (i.e., by providing repeatability and good lighting conditions and eliminating artifacts). In terms of software, the key is to ensure an appropriate level of product classification effectiveness (defect detection), while maintaining high computing performance. For high-performance production lines, increasing the accuracy level even by half a percent can be a very significant profit for the manufacturer. Furthermore, the software should be accordingly designed to reduce the risk of human error and to make its operation as simple as possible. It is related to the reduction of image processing parameters that should be set for proper product control, as well as the possibility of easy adaptation of existing algorithms or settings to other formats of a given product or other production lines. Therefore, the classical methods of image processing are increasingly being replaced by methods of deep-based neural networks [9,10,11,12], and the necessity of manual parameters setting in classic image processing algorithms turns into the model training process, i.e., collecting the appropriate number of examples of tested products (as well as examples with defects in the case of supervised learning methods). Collecting a sufficient number of examples with defects or faults can be cumbersome in some real cases. Imbalanced datasets can in consequence deteriorate the accuracy of the classification model. To overcome this obstacle, techniques such as data augmentation and/or data generation with generative adversarial networks can be used [13,14].

Machine learning methods and neural networks provide fast and accurate image classification results. For this purpose, neural network models pre-trained on the ImageNet [15] and the transfer learning technique [16] can be used to adjust the classification layer to specific needs. To efficiently train large models, some modifications of the learning algorithms can be used [17]. Due to the increasing efficiency of production lines, as well as the production of various formats on the same line (e.g., different heights of bottles, colors of caps, printing, etc.), it is reasonable to use the most lightweight models with less complex architecture which ensures an equally high level of classification and requires smaller training sets [18]. To achieve this, knowledge about the examined objects and conditions of image acquisition as well as the development of dedicated algorithms for image preprocessing can be utilized [19]. Image preprocessing based on the knowledge about the optical system and the characteristics of the tested object allows simplifying the models, obtaining better classification results and shorter classification and training time.

The most common practice in packaging liquid products in the food industry is pouring into polyethylene terephthalate (PET) and glass bottles. High production efficiency and wear of elements of filling and capping machines cause the occurrence of cap defects, such as discoloration, cracks, incorrectly attached safety rings, and cap skews. These defects may occur around the whole closure, making them difficult to detect and remove from the production line. If a bottle with a defective closure reaches the customer, the manufacturer is at risk of return of an entire batch of products and financial and reputational loss. Likewise, manufacturers strive to increase the performance and speed of their production lines (in the case of liquid products, even up to 120,000 pieces per hour), which results in an increased occurrence of defects. It is connected with unplanned production interruptions relating to the detection of defects and failure of production lines which result in even greater financial losses. Thus, there is a need for drug and beverage manufacturers to install vision quality control systems that ensure high-level defect detection for highly efficient production.

The hardware solutions are based mainly on increasing the number of cameras to observe the cap in a range of 360 degrees and from the top. Cameras directed on the side of the cap allow side surface defects detection, while the camera directed on the top allows the top surface defects detection. The decision on whether the bottle should be rejected from the production line can be based on the combination of classification results from all cameras. Other solutions include mechanical rotation of the bottles and analysis of images taken from one point on the side of the cap. Images obtained using this approach may be difficult to analyze due to changing lighting conditions, shadows, glare, and different closure colors. Image analysis can be based on classical methods as well as on neural networks [5,10,20].

Solutions based on deep neural networks may provide better results in defect detection and classification, but the network training process may be too long, especially for lines on which formats often change. Moreover, the use of the currently most effective neural network models (Xception [21], Vgg [22], and ResNet50 [23]) for images from each camera may significantly reduce the efficiency of quality control and, consequently, limit the fields of application of these methods for high-speed production lines.

Ensuring the appropriate accuracy of classification while maintaining efficiency requires a combination of hardware and software solutions. In our solution, we use a dedicated optical system that allows observation of the cap in the range of 360 degrees and at the top with only one camera and one image (Inspect360+ [24]). The hardware system is also designed to avoid problems with lighting and reflections to facilitate image data analysis. This architecture allows assessment of the quality of the closure by analyzing only one image, taken under very reproducible conditions. The image of each product is similar and the defects are visible and easy to distinguish. The system has implemented an algorithm for image preprocessing, which is based on the optical system calibration method and allows reduction of the resolution of the original image resolution. The preprocessing algorithm provides an image which is easier in visual interpretation for the neural network and for a human (which may be important for employees of production and maintenance departments). The method is characterized also by significantly better efficiency and effectiveness of defect detection. Furthermore, due to the calibration of the optical system, it is possible to remove the influence of imperfections of the optical system. Thus, similar defects have the same appearance regardless of their location on the tested object and the use of the transfer learning technique for implementation of the system on other production lines is feasible. The images are classified using the developed lightweight model of the Convolutional Neural Network (CNN) network.

The aim of this work is to develop a pipeline for images from the liquid closure quality control system processing, which, in combination with a dedicated optical system, can ensure high efficiency of prediction and training as well as high efficiency of detection and classification of defects. Based on the knowledge of the optical system and the tested objects, dedicated image preprocessing algorithms and a lightweight neural network model were developed. The obtained classification results, as well as the prediction and training times of the models, were compared with the models achieving the best results on the ImageNet. The impact of the preprocessing stage on the results was also evaluated.

## 2. Materials and Methods

### 2.1. The Dataset

**Figure 1:**
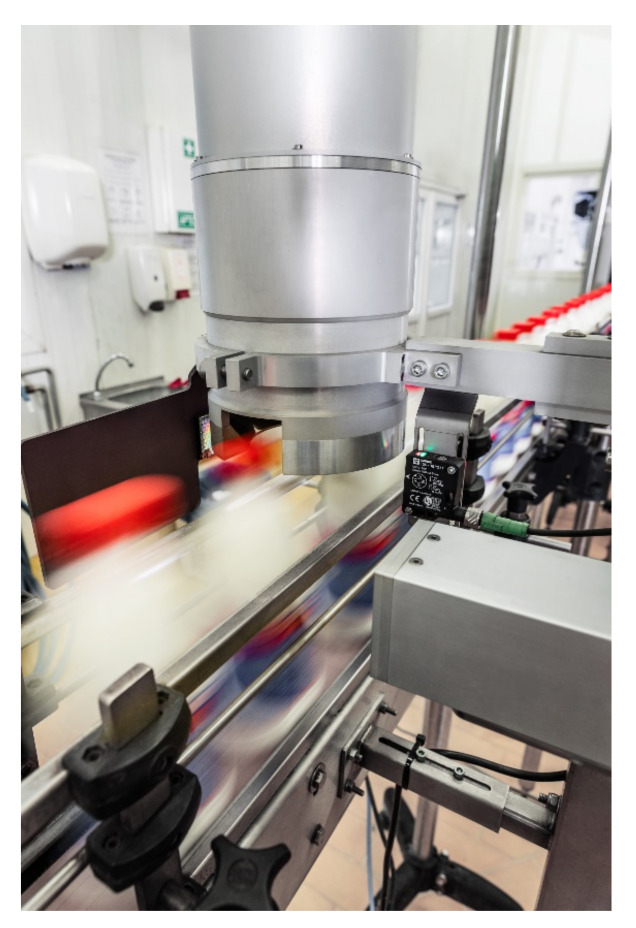
Inspect360+ head installed in the production line.

The Inspect360+ system installed on the production line for bottling milk was used. Image processing algorithms and neural network models were implemented in Python using the Tensorflow [25] and OpenCV [26] libraries. The dataset consists of grayscale images with a resolution of 600 × 600 pixels. All images were collected using the Inspect360+ PET bottle closure quality control system installed on the milk bottling production line. The production line and the system are presented in Figure 1. Due to the low frequency of occurrence, in the first stage of the work, the defects were prepared manually based on an interview with line operators about the most common defects. After the initial development of the classification models, the system recorded examples marked as defects during a few weeks. Then, the dataset was completed with actual examples of defects from the production line (after visual verification). The defects were divided into:

- good cap
- faulty cap
- dirty cap
- missing cap
- unknown

**Figure 2:**
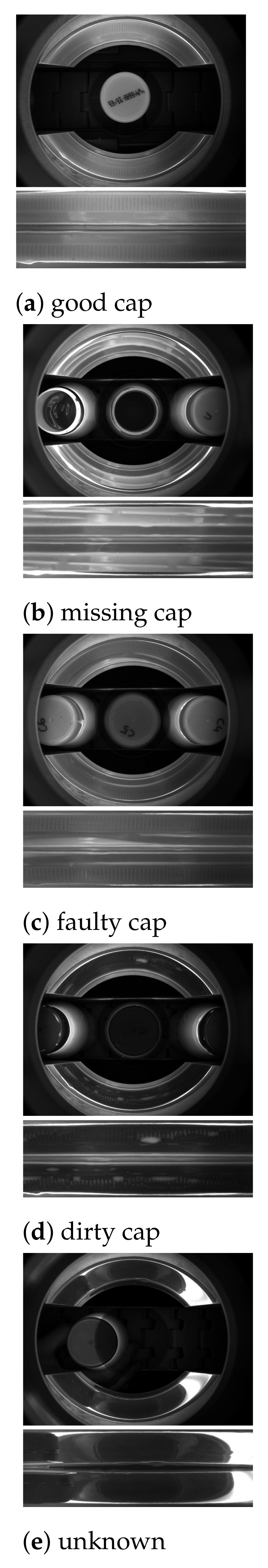
Example original images acquired with Inspect360+ and corresponding images after calibration procedure for each class.

Side images of each class and corresponding images acquired by the Inspect360+ system are presented in Figure 2 Class “good cap” signifies samples without any faults or dirt. Class “faulty cap” signifies samples with a skew cap or incorrectly attached safety ring. Class “dirty cap” was added after the stage of collecting data on the actual production line and includes examples that are not defects but significantly different from the examples of “good cap” class. Dirt is usually a small amount of milk that remains on the pouring and closing machines, which then sediments on the caps. Formation of a “dirty cap” class allows the production manager to obtain additional information about a potential problem on the production line and at the same time facilitates the process of training the neural network model. The “missing cup” class signifies samples without a cup. The “unknown” class was added to cover the situation where the image was triggered at a random moment. It contains images with artifacts, e.g., images of an empty production line or incorrectly triggered images.

The final dataset was divided into training, validation, and test datasets. The number of samples of each class within each set is presented in Table 1.

### 2.2. Calibration Procedure

Images in the Inspect360+ system are taken by the camera placed above the bottle cap. The cap is recorded in the images after being transformed by the optical system (as shown in Figure 2). Images obtained in the presented way are difficult in visual interpretation and the available image resolution is not optimally used. To improve detection, an image preprocessing algorithm involving the calibration of the optical system Inspect360+ was developed.

**Figure 3:**
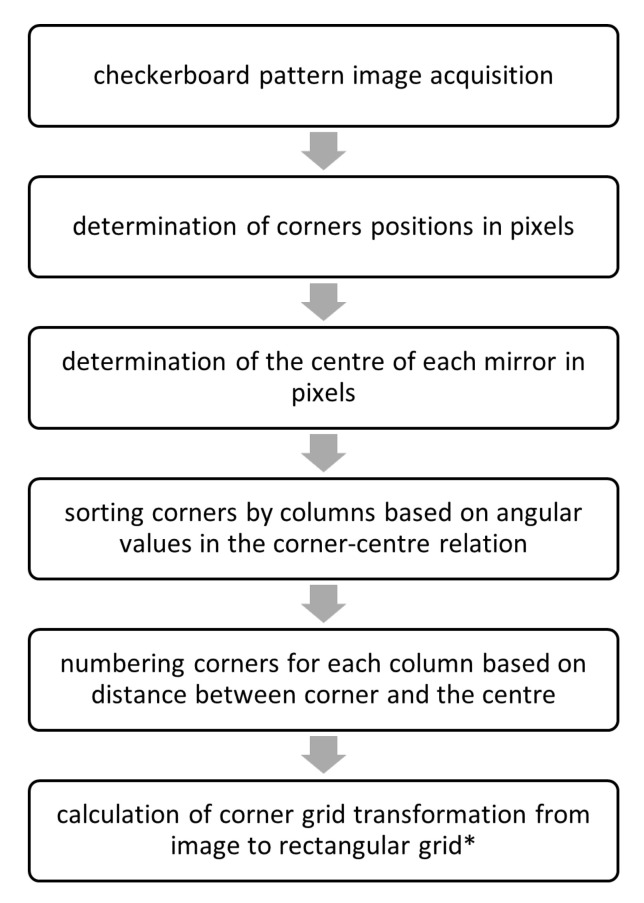
The scheme of the calibration procedure. * At this stage, output resolution is defined, and there is no need to change the resolution at other stages.

The calibration method is based on a picture of a checkerboard pattern with a known geometry using the Inspect360+ system camera. The checker pattern should be dense to accurately represent the geometry of the optical system and compensate for its technological imperfections. However, a too dense grid may decrease the accuracy of the determination of corners and the accuracy of the calibration may be reduced. The image of a pattern is taken to obtain an image of an object deformed by the optical system with known geometry. The obtained image is analyzed and the transformation generated by the optical system is calculated. Furthermore, the transformation is applied to the images of the analyzed products. This results in obtaining images that are easier for visual analysis and the fully utilize available resolution (pixels that do not contain information about the defects of the tested surface are removed). Moreover, if the same quality control system is installed on other production lines, the calibration process will allow the image classification models to be insensitive to the imperfections of optical system components, which will facilitate the use of the transfer learning technique or even allow the classification model to be copied between devices, which will shorten the installation time of new devices (no need to train the model). Likewise, the correction of imperfections of optical system components results in the appearance of similar defects in different areas of the tested object in the same way, which should positively affect the process of teaching the classification model. The calibration procedure is presented in Figure 3.

**Figure 4:**
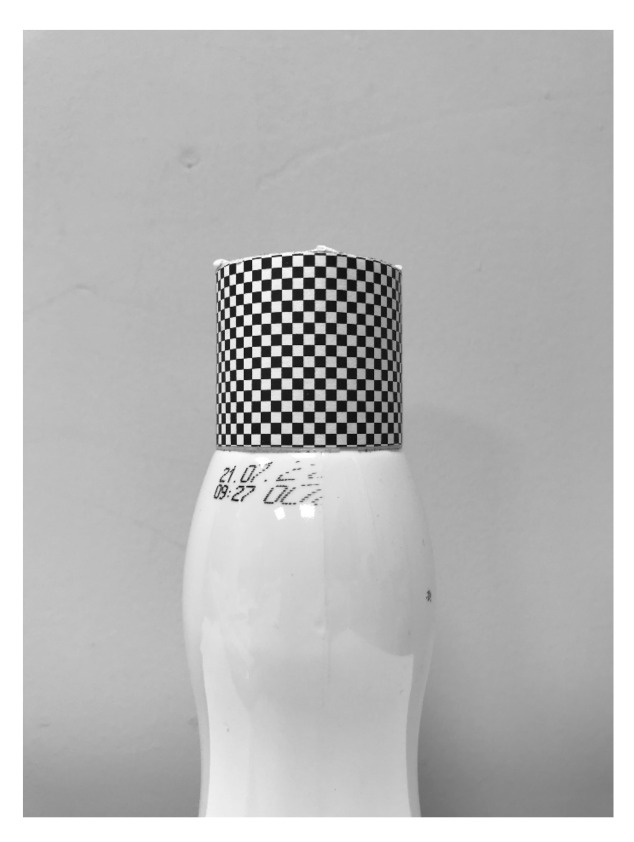
Calibration pattern wrapped around a cap; the sample was used for calibration of the system.

The checkerboard pattern is placed around the closure (cap) of the controlled product. Thus, the transformation determined in the calibration procedure is adapted to the geometry of the controlled object (Figure 4).

**Figure 5:**
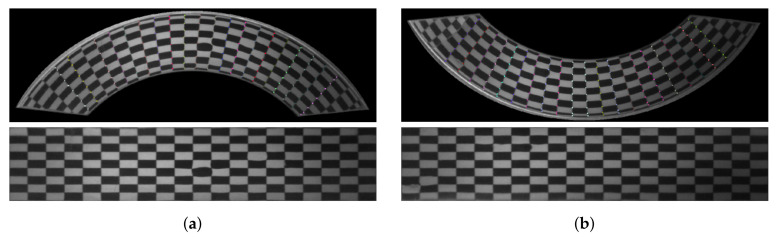
(**a**) The upper mirror with the applied mask and corresponding image of the upper mirror after transforming to a rectangular grid; and (**b**) the lower mirror with the applied mask and corresponding image of the lower mirror after transforming to a rectangular grid.

Then, an image of the calibration pattern is taken and the positions of the checkerboard corners are determined using the Harris algorithm [27] implemented in OpenCV library [26]. In the next step, the center of each mirror is determined in pixels to facilitate numbering of the checkerboard grid. The image of the calibration pattern taken by the system Inspect360+ is shown in Figure 5a. It shows eliminating the background mask and the marked corners detected in the image. Numbering of the detected corners is divided into two steps. In the first step, the corners are sorted by columns based on angular value (in relation to the mirror’s center). In the second step, the corners in each column are sorted based on the distance to the mirror’s center. The corners numeration expressed in colors corresponds to the numeration on the real, non-deformed checkerboard presented with the adopted coordinate system in Figure 5a. The coordinates of the corners on the non-deformed board can be chosen arbitrarily, but the width and height of the “straighten” board should approximately correspond to the height and width of the board deformed by the optical system. If the checkerboard is smaller, then some of the data recorded by the camera will be lost (lowering the resolution). If the checkerboard is larger than the original, interpolation algorithms will be used, which will not add any information to the image, but only extend the processing and classification time of images. In the next step of the procedure, the transformation between the coordinates of the corners recorded by Inspect360+ and the coordinates in the adopted coordinate system for the non-deformed checkerboard is determined. Then, the mapping function in the *x* and *y* directions is determined based on the detected coordinates of the corners in the original image. The mapping function is used to “straighten” the deformed image. The result of the simple remapping routine is presented in Figure 5b.

**Figure 6:**
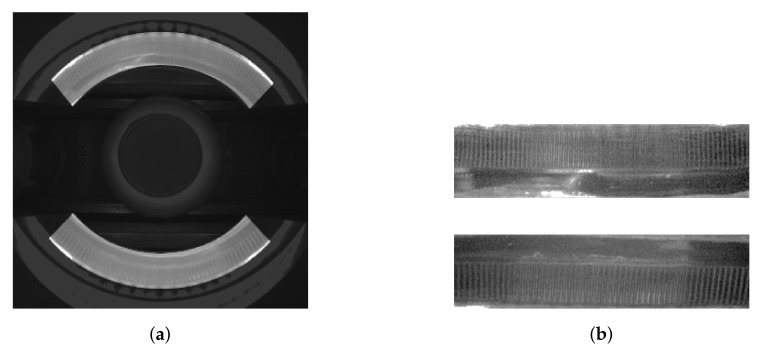
(**a**) The original image acquired with Inspect360+, where the area of the image that represents the cap is highlighted; and (**b**) the upper half and lower half of the cap transformed with the calibration procedure.

As shown in Figure 6, the transform matrix is determined independently for the two image halves. This approach is due to the convenience of using the remapping function and the image interpolators embedded in it. The output is then two images: for the upper half and the lower half of the mirror. In the presented study, a chessboard with 3 mm spacing was used. The mesh was wrapped around a 40 mm diameter cap. The size of images representing the halves of the original image (bottom and top) is 74 × 640 pixels.

### 2.3. Deep Learning Models

**Figure 7:**
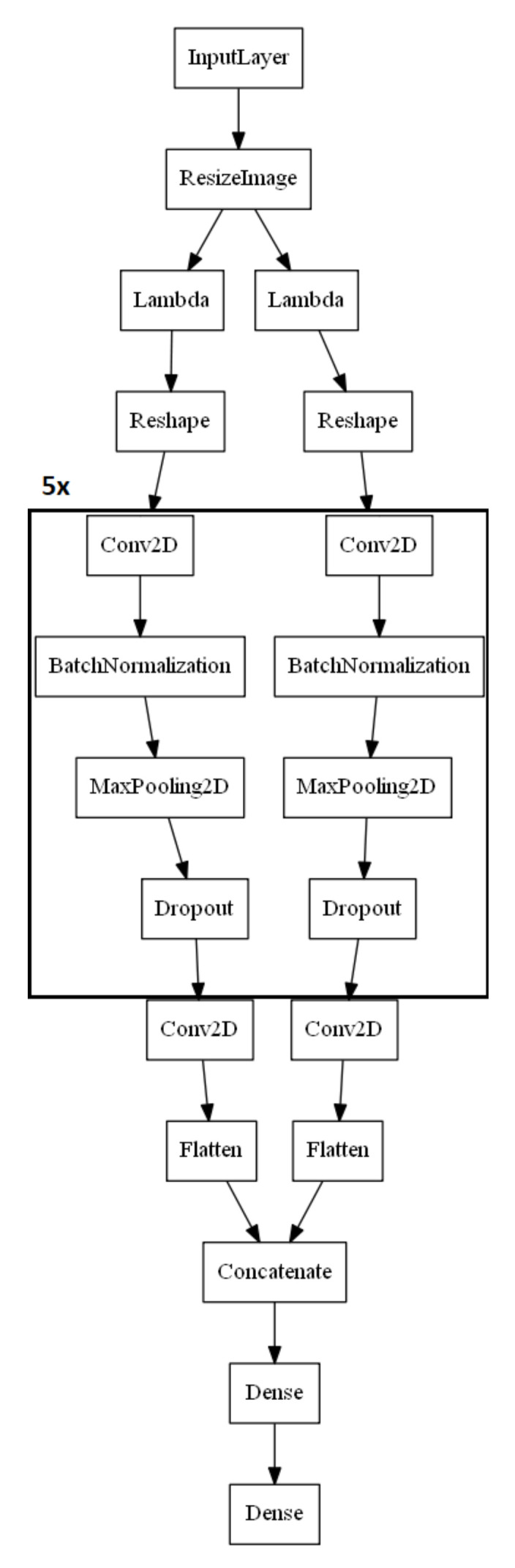
The architecture of CustomKSM model.

In the presented research, well-known, lightweight models, obtaining the best results in benchmarks on the ImageNet and a model based on its architecture were used. Light models were chosen due to their intended use in industrial conditions, on high-speed production lines. In particular, the following models were used: Resnet50 [23], Xception [21], and MobileNetV2 [28]. The architecture of the developed model (CustomKSM) is presented in Figure 7. In the CustomKSM model, images are analyzed in separate channels. After feature extraction in separate channels, the classification in the fully connected layer is conducted. Channel separation for both images allows matching the filters to the characteristics of the images of each of the halves. The model uses a strong regularization through dropout layers due to the potentially large variability of product images taken on the production line.

**Figure 8:**
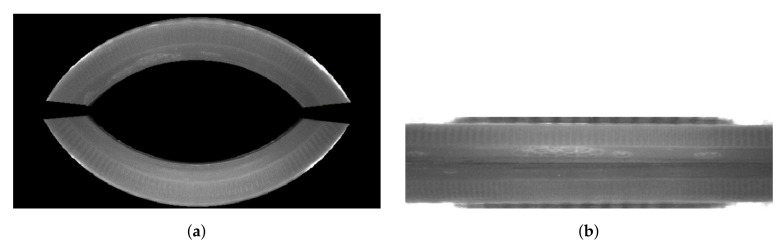
Image of side surface of a cap: (**a**) masked image after cutting out unnecessary background; and (**b**) image that is a combination of mirrors images after calibration.

The CustomKSM model was optimized with respect to the selection of hyperparameters (e.g., the number of convolutional layers and filters in each layer, the dropout parameter, and the number of neurons in the Fully Connected layer). Models with weights pre-trained on the ImageNet were fine-tuned to classify cap defects based on the collected dataset, while the CustomKSM model was trained from scratch. To investigate the effects of preprocessing, pre-trained models were trained on both original and calibrated images. In the case of training on the original images, an area containing no information about the tested object (i.e., the conveyor belt, the Inspect360+ system housing, and the upper part of the cap) was masked and partially cut out in these images to prevent training based on false features (Figure 8).

#### Training and Evaluation

The tested models were trained using the Tensorflow library. Training parameters are presented in Table 2.

**Figure 9:**
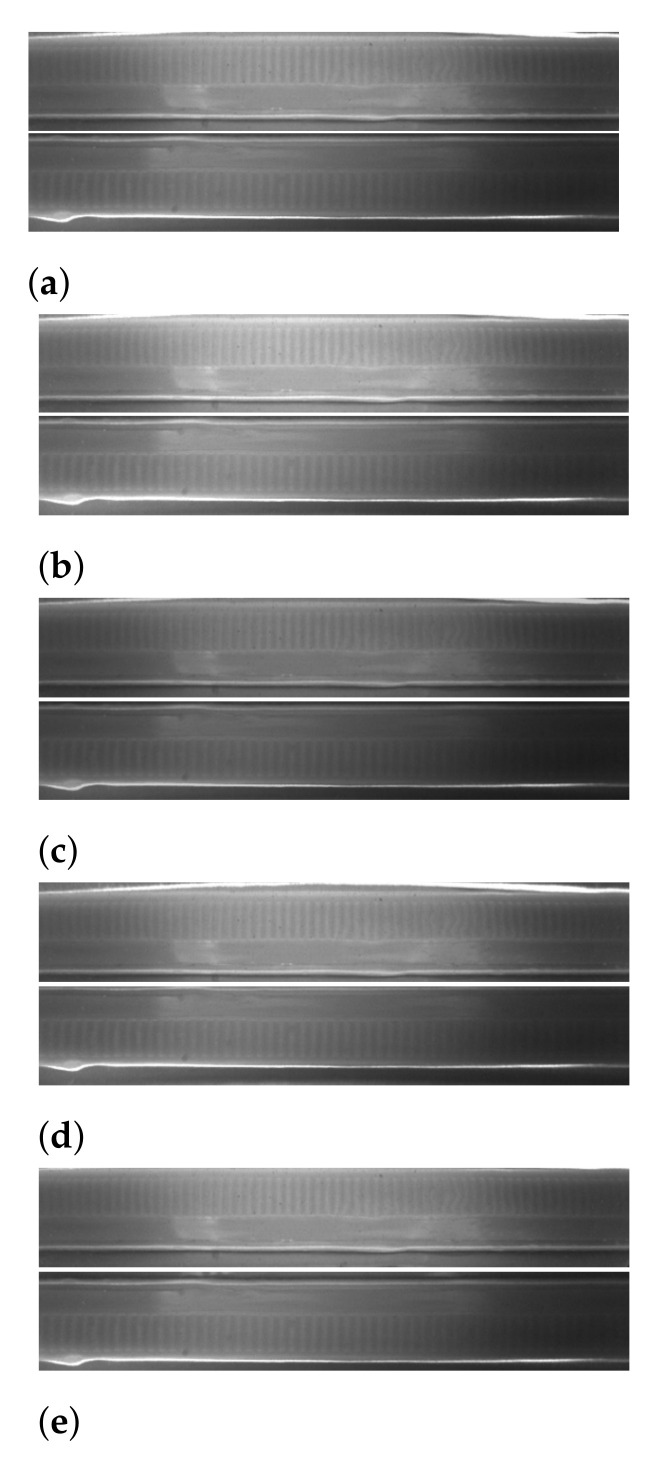
Data augmentation: original image after calibration (**a**); brightness changes of 0.8 and 1.2 (**b**,**c**); and shifts on y-axis of −5 px and +5 px (**d**,**e**).

Due to the high efficiency of the production line and production volumes, the analyzed examples are highly variable. The capping machine can cause any defect, and the defects can be very different from each other and placed in the lower part of the cap. Differences in the analyzed examples are also caused by the position of the bottle in relation to the camera: in the direction of the transporter’s movement as a result of the moment of cutting the optical barrier which is triggering the camera, and in the perpendicular direction due to the width of the transporter. Additionally, the bottles (blown from pre-fab) and caps may vary in height within some tolerance. Furthermore, images may differ in the intensity of lighting as the system is running due to the consumption of light-emitting diodes (LED) illuminator. Therefore, the obtained dataset (approximately 7000 examples) is small compared to the number of examples that can be analyzed in 24 h (up to 200,000 bottles). To obtain the best possible generalization of the models, data augmentation was implemented in the model learning stage. However, because of the rigid nature of the Inspect360+ system with its fixed camera field of view, traditional augmentation methods fail. Transformations such as image shift, cropping, and rotation do not imitate real data variation. In the case of transformation on the input image, the image cannot be successfully calibrated. Calibration is determined for a system in which its parts do not shift. The real diversity of the data is expressed by the variable height of the bottles, the change of which is observed on both mirrors simultaneously. This is a requirement for custom data augmentation in which it is necessary to imitate the height difference in calibrated images. It was implemented in the form of cropping calibrated images with a predetermined output resolution and vertical shifts in the form of an offset. This shift has the same value for both images, but it has the opposite direction. The upward visual shift in the upper mirror is visible as a downward shift in the lower mirror and vice versa. Besides, the augmentation of the brightness of calibrated images was implemented, which also meets the requirements for differentiation of production data. The image after calibration and examples of the same image after applying the augmentation algorithm are presented in Figure 9. The adopted ranges of parameters for the data augmentation function are presented in in Table 3.

The models were evaluated on the basis of the accuracy of assigning examples to appropriate classes.

The column “Modes” indicates whether the data were calibrated (preprocessed) and whether the images were analyzed in separate (Separated) or connected (Concat) channels after calibration. The inference times presented in Table 4 are the combined times of preprocessing (where it was applied) and inference. Detailed analyses of the obtained results are presented in the following subsections.

### 2.4. Comparison of the Accuracy of the Selected Models

**Figure 10:**
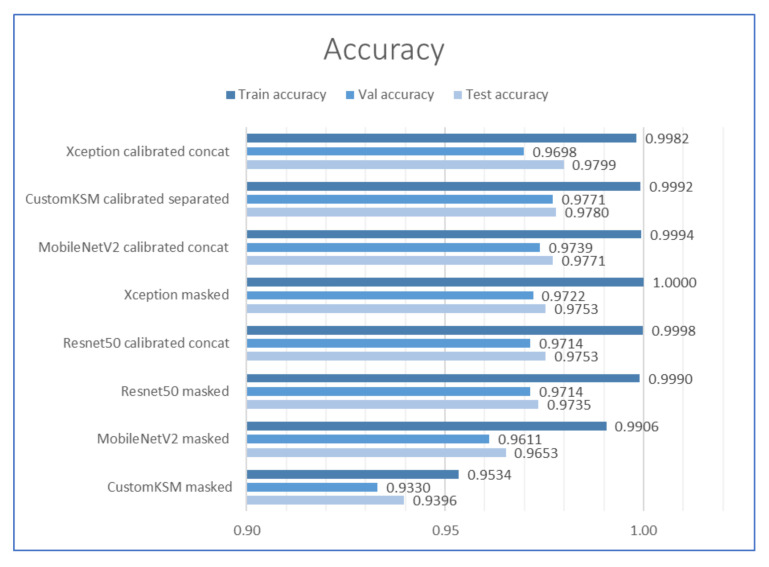
Accuracy of the tested models for train, val and test datasets, the higher the better.

Accuracy of each of the tested models is presented in Figure 10. All models achieved similar accuracy after training on the full dataset. The highest accuracy for the test set was achieved by the Xception model (0.9799) operating on the images after calibration. The CustomKSM model obtained a slightly lower result (0.978). For most models, the accuracy on the test set was slightly higher than on the validation set, which may indicate overfitting of the models. Analysis of the obtained results showed that, for each model, calibration improves the accuracy of the test set. In industrial applications for highly efficient production, an improvement of even 1% can already make a big difference to maintenance and production departments. As expected, a particularly large increase in accuracy was observed for the minimalist model of the developed architecture (CustomKSM masked). In the case of such models, the appropriate preprocessing of images, which enhances the features that differentiate individual classes, plays an important role. Comparison of accuracy showed that, with the accurate preprocessing of images (based on the knowledge of the optical system and image acquisition conditions), it is possible to achieve classification results with a lightweight model at least at the same level as with the use of large models, trained on the ImageNet.

### 2.5. Comparison of the Inference Times

**Figure 11:**
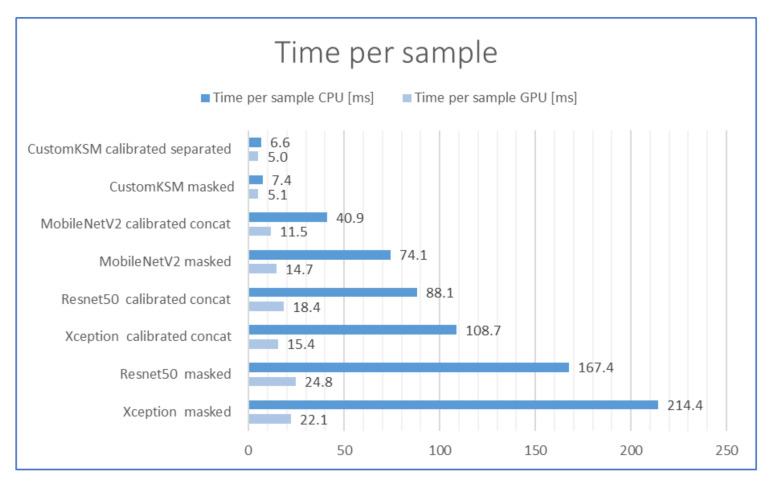
Inference times for the tested models in milliseconds, the lower the better.

Comparison of inference times (including preprocessing in case of calibrated images) is presented in Figure 11. Inference time for the CustomKSM model is significantly shorter than for any other model tested. It is 2.5 times shorter than the second-best MobilnetV2 model on graphics processing unit (GPU), and it is eight times shorter on central processing unit (CPU). The time of processing and inference is especially important in industrial installations with high-speed production lines. The obtained results for the CustomKSM model allow implementation even on ultra-fast production lines with capacities above 70,000 pieces per hour. To obtain sufficient performance, the GPU is not needed, and the CPU could have even lower performance (which is also important in terms of the cost of the entire quality control system).

For each of the remaining models, it can also be observed that the calibration process reduces the inference time by approximately two times. This is the effect of reducing the resolution of the input images. The calibration allowed for more effective use of the available resolution of the original image.

### 2.6. Comparison of the Training Times and Training Performance

**Figure 12:**
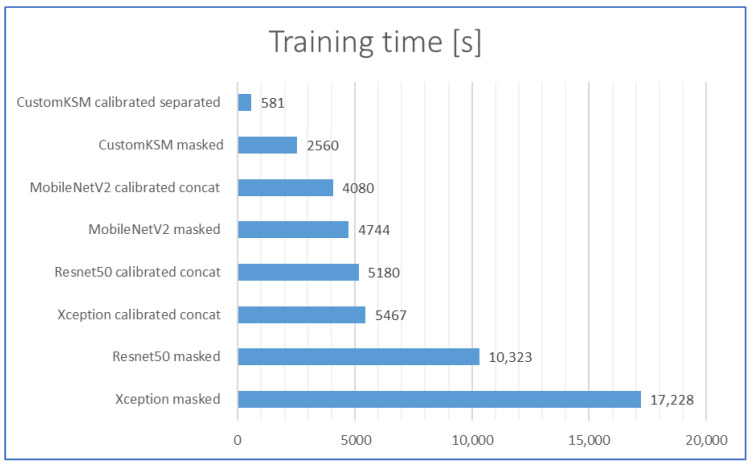
Training. times of the tested models on CPU (full dataset), the lower the better.

Training times for each of the tested models on CPU are presented in Figure 12. As one can observe in Figure 12, CustomKSM model can be trained within 5 or 6 min on a relatively big dataset. Although in laboratory work the differences in training times are not very significant, in the case of industrial applications, they become more important. In the industrial environment, conditions may change as a result of factors such as vibration, unintentional impact on the conveyor or quality control system, dust on the optical system components, change of color, or tolerance of semi-products such as caps or bottles. This can significantly decrease the accuracy of classification of the developed and learned models. Nevertheless, the improvement of performance can be obtained by “post-training” the model. Because of such short training times, post-training can be performed even on the CPU of the production computer, directly at the production line (i.e., by the production line operators after appropriate training). Moreover, short times of model learning can be important in the case of production lines with frequent changes in the produced formats (reformatting time and settings changes are important parameters in assessing the suitability of machines on the production line). As in the case of inference times, the training times for the models with preprocessing are about two times shorter, which is a result of the lower resolution of the input images.

## 3. Discussion

Unlike general neural networks models that can be used for many different tasks [15], the presented approach of designing of the classification model requires a priori knowledge of the optical system and/or inspected object. Preprocessing is dedicated to a particular application for maximum utilization of the available resolution of the image and enhancement of the distinctive features. In the case of production lines of lower capacities, it might be more convenient to use standard neural nets pre-trained on Imagenet, as it would shorten implementation time of the quality inspection system. However, even when the standard networks are used, the developed calibration method allows improving the classification accuracy (by up to 1% in the presented case), which is essential in industrial applications.

An additional advantage of calibration is the standardization of images from the quality inspection system between its implementations on different production lines in various production plants. Hence, it is possible to use the same model to extract features in other plants, which significantly reduces the implementation time of the quality inspection system. Furthermore, all installed quality inspection systems may benefit from subsequent implementations of the same system. Calibrated images expand a common database and help to achieve better accuracy.

The presented quality inspection system can be easily adapted to other tasks such as inspection of glass vials in pharmaceutical industry [29] or bottles closings in chemical and cosmetics industry. It is planned to test the accuracy of the developed model and calibration method of the Inspect360 + system in other industries. The research will take into account the transfer learning technique [9] to use the weights trained on the caps of PET bottles. The main limitation in future applications of the system might be the diameter of inspected objects, as increasing the diameter of the measuring head would significantly increase the costs of the system.

Another direction of the future works is to overcome the problem with imbalanced datasets due to the limited number of real examples of faulty products. In this case, it is planned to use generative adversarial networks (GAN) [13] to expand the dataset. Using GANs to generate new data from known examples might produce better results than data augmentation [30] as simple data augmentation procedures do not represent actual variations of images after calibration. GANs have already been used for data generation in industrial applications of neural networks and show promising results [14].

## 4. Conclusions

The article presents the application of deep neural network methods for the detection and classification of defects in closures of liquid packaging on production lines. To solve the practical problems related to the use of overtrained networks on the ImageNet (computation power requirements and training and inference times), a very lightweight network architecture is proposed, which, in combination with the developed method of optical system calibration, allows obtaining at least the same accuracy compared to known models and several times shorter inference and training times. On the example of the quality control of closures for liquids, it is proven that, in industrial applications of neural networks, much better parameters can be obtained by basing data preprocessing on the knowledge about the optical system. In addition to classification accuracy gains, dedicated image preprocessing enables standardization of data obtained on similar production lines and shorten the time needed to implement quality control system.

The presented system can be used for quality inspection tasks in other industries such as inspection of glass vials closures in the pharmaceutical industry, bottle caps in the brewing industry or cylindrical containers closures in the chemical or cosmetics industry.

## Figures and Tables

**Table 1:** Number of samples in each subset of the dataset.

Class	Training	Validation	Test
good cap	2058	515	460
faulty cap	794	198	176
dirty cap	1830	458	410
missing cap	186	46	42
unknown	25	6	5

**Table 2:** Training parameters used in the training process.

Parameter	Value
learning rate	0.001 *
cost function	categorical_crossentropy
optimizer	adam
metrics	categorical_accuracy
epochs	100

* learning rate drops 0.5 each 30 epochs.

**Table 3:** Data augmentation ranges.

Augmentation	Range
Shift y	(−5, 5)
Brightness	(0.8, 1.2)

**Table 4:** The results of the models’ evaluation. The best results are bolded.

Model	Modes		Weights	Accuracy			Training Time [s]	Inference Time [ms]	
	Calibrated	Input		Train	Val	Test		CPU	GPU
CustomKSM	Yes	Separated	From scratch	0.9992	**0.9771**	0.978	**348**	**5.1**	**4.4**
CustomKSM	No	-	From scratch	0.9534	**0.9330**	0.9396	**2560**	**7.4**	**5.1**
MobileNetV2	No	-	ImageNet	0.9906	0.9611	0.9653	4744	74.1	14.7
MobileNetV2	Yes	Concat	ImageNet	0.9994	0.9739	0.9771	4080	40.9	11.5
Resnet50	No	-	ImageNet	0.999	0.9714	0.9735	10,323	167.4	24.8
Resnet50	Yes	Concat	ImageNet	0.9998	0.9714	0.9753	5180	88.1	18.4
Xception	No	-	ImageNet	1	0.9722	0.9753	17,228	214.4	22.1
Xception	Yes	Concat	ImageNet	0.9982	0.9698	**0.9799**	5467	108.7	15.4

## Data Availability

The data presented in this study are available on request from the corresponding author. The data are not publicly available because it contains images acquired by devices working at our clients’ premises.
